# Crystal structure of 2-[(*E*)-2-(2-chloro­benzyl­idene)hydrazin-1-yl]-4-phenyl-1,3-thia­zole

**DOI:** 10.1107/S1600536814016298

**Published:** 2014-08-01

**Authors:** Joel T. Mague, Shaaban K. Mohamed, Mehmet Akkurt, Alaa A. Hassan, Mustafa R. Albayati

**Affiliations:** a Department of Chemistry, Tulane University, New Orleans, LA 70118, USA; b Chemistry and Environmental Division, Manchester Metropolitan University, Manchester M1 5GD, England; c Chemistry Department, Faculty of Science, Mini University, 61519 El-Minia, Egypt; d Department of Physics, Faculty of Sciences, Erciyes University, 38039 Kayseri, Turkey; e Kirkuk University, College of Science, Department of Chemistry, Kirkuk, Iraq

**Keywords:** crystal structure, 1,3-thia­zole, hydrogen bonding, hydrogen-bonded dimers

## Abstract

The asymmetric unit of the title compound, C_16_H_12_ClN_3_S, contains two independent mol­ecules whose conformations differ primarily in the orientations of the phenyl and chloro­benzene rings with respect to the thia­zole ring. In the first mol­ecule, the dihedral angles are 3.0 (1) and 9.2 (1)°, respectively, for the phenyl ring and the chloro­benzene ring, while in the second mol­ecule, the corresponding angles are 18.6 (1) and 23.4 (1)°. In the crystal, the two independent mol­ecules are associated *via* complementary N—H⋯N hydrogen bonds into a dimer. These dimers are associated through weak C—H⋯Cl and C—H⋯S inter­actions into supra­molecular chains propagating along the *a*-axis direction.

## Related literature   

For pharmaceutical properties of thia­zole derivatives, see: Siddiqui *et al.* (2011 ▶, 2009 ▶); Bakris *et al.* (2004 ▶); Little *et al.* (2005 ▶). For the synthesis of the title compound, see: Mohamed *et al.* (2013 ▶).
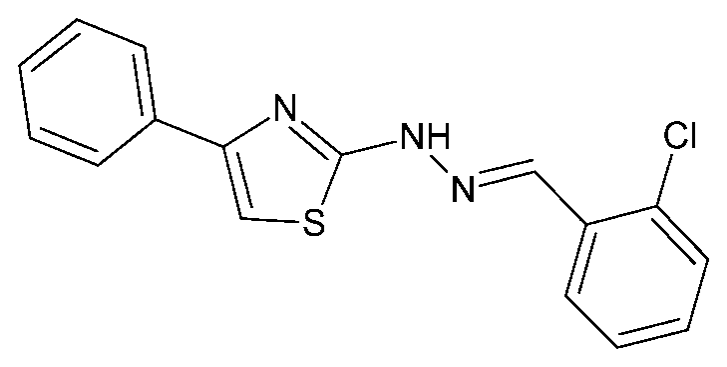


## Experimental   

### Crystal data  

C_16_H_12_ClN_3_S
*M* *_r_* = 313.80
Orthorhombic, 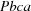

*a* = 16.981 (4) Å
*b* = 8.1081 (17) Å
*c* = 41.660 (9) Å
*V* = 5736 (2) Å^3^
*Z* = 16
Mo *K*α radiation
μ = 0.41 mm^−1^
*T* = 150 K
0.22 × 0.22 × 0.05 mm

### Data collection  

Bruker SMART APEX CCD diffractometer
Absorption correction: multi-scan (*SADABS*; Bruker, 2013 ▶) *T*_min_ = 0.80, *T*_max_ = 0.98
99305 measured reflections
7415 independent reflections
5577 reflections with *I* > 2σ(*I*)
*R*_int_ = 0.086

### Refinement  

*R*[*F* ^2^ > 2σ(*F* ^2^)] = 0.044
*wR*(*F* ^2^) = 0.109
*S* = 1.04
7415 reflections
379 parameters
H-atom parameters constrained
Δρ_max_ = 0.42 e Å^−3^
Δρ_min_ = −0.33 e Å^−3^

### 

Data collection: *APEX2* (Bruker, 2013 ▶); cell refinement: *SAINT* (Bruker, 2013 ▶); data reduction: *SAINT*; program(s) used to solve structure: *SHELXTL* (Bruker, 2013 ▶); program(s) used to refine structure: *SHELXL2014* (Sheldrick, 2008 ▶); molecular graphics: *DIAMOND* (Brandenburg & Putz, 2012 ▶); software used to prepare material for publication: *SHELXTL*.

## Supplementary Material

Crystal structure: contains datablock(s) global, I. DOI: 10.1107/S1600536814016298/xu5803sup1.cif

Structure factors: contains datablock(s) I. DOI: 10.1107/S1600536814016298/xu5803Isup2.hkl

Supporting information file. DOI: 10.1107/S1600536814016298/xu5803Isup3.cml

**Fig. 1.**
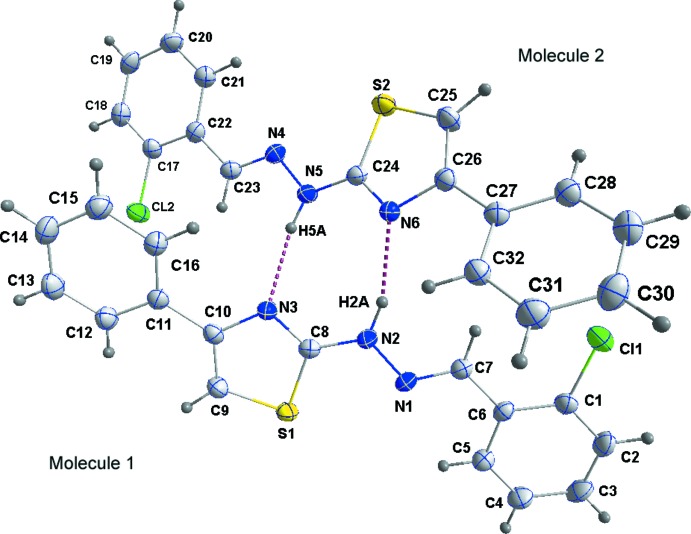
The asymmetric unit with the complementary N—H···N hydrogen bonds shown as dotted lines. Ellipsoids are drawn at the 50% probability level.

. DOI: 10.1107/S1600536814016298/xu5803fig1.tif
The asymmetric unit with the complementary N—H⋯N hydrogen bonds shown as dotted lines. Ellipsoids are drawn at the 50% probability level.

CCDC reference: 1013753

Additional supporting information:  crystallographic information; 3D view; checkCIF report

## Figures and Tables

**Table 1:** Hydrogen-bond geometry (Å, °)

*D*—H⋯*A*	*D*—H	H⋯*A*	*D*⋯*A*	*D*—H⋯*A*
N2—H2*A*⋯N6	0.91	2.02	2.901 (2)	163
N5—H5*A*⋯N3	0.91	2.05	2.946 (2)	166
C9—H9⋯S1^i^	0.95	2.97	3.696 (2)	134
C25—H25⋯Cl1^ii^	0.95	2.92	3.720 (2)	143

Symmetry codes: (i) 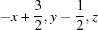; (ii) 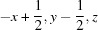.

## supplementary crystallographic information

### S1. Comment

Thiazole containing compounds have been reported to possess large number of biological properties (Siddiqui *et al.*, 2011; Siddiqui *et al.*, 2009). Sulfathiazol (antimicrobial drug), Ritonavir (antiretroviral drug), Abafungin (antifungal drug), Bleomycine and Tiazofurin (antineoplastic drug) are common drugs with thiazole-based structures. Alagebrium (formerly known as ALT-711) is also a thiazolium salt which was the first drug used for breaking the protein crosslinks caused by advanced glycation endproducts (AGEs). Through this effect Alagebrium is designed to reverse the stiffening of blood vessel walls that contributes to hypertension and cardiovascular disease (Bakris *et al.*, 2004; Little *et al.*, 2005). İn this context and as part of our study in synthesis of potential bioactive heterocyclic molecules, we report the synthesis and crystal structure of the title compound.

There are two independent molecules of the title compound in the asymmetric unit whose conformations differ primarily in the orientations of the phenyl rings with respect to the thiazole ring. For molecule 1, the dihedral angles are 3.0 (1) and 9.2 (1)°, respectively, for rings C1–C6 and C11–C16 while for molecule 2 the corresponding angles are 18.6 (1) and 23.4 (1)°.

In the crystal structure, the two independent molecules are associated *via* complementary N—H···N hydrogen bonds (Fig. 1 and Table 1). These pairs are associated into chains running along the *a* axis through weak C—H···Cl interactions (Table 1) and weak C—H···S interactions (Table 1).

### S2. Experimental

The title compound has been prepared according to our reported method (Mohamed *et al.*, 2013). Colourless crystals suitable for X-ray diffraction have been obtained by crystallization of the crude product (I) from ethanol.

### S3. Refinement

H-atoms attached to carbon were placed in calculated positions (C—H = 0.95 Å) while those attached to nitrogen were placed in locations derived from a difference map and, following initial independent refinement to verify their presence, their coordinates were adjusted to give N—H = 0.91 Å. All were included as riding contributions with isotropic displacement parameters 1.2 - 1.5 times those of the attached atoms.

### Crystal data

**Table:**

C_16_H_12_ClN_3_S	*D*_x_ = 1.454 Mg m^−^^3^
*M**_r_* = 313.80	Mo *K*α radiation, λ = 0.71073 Å
Orthorhombic, *P**b**c**a*	Cell parameters from 9986 reflections
*a* = 16.981 (4) Å	θ = 2.6–28.7°
*b* = 8.1081 (17) Å	µ = 0.41 mm^−^^1^
*c* = 41.660 (9) Å	*T* = 150 K
*V* = 5736 (2) Å^3^	Plate, colourless
*Z* = 16	0.22 × 0.22 × 0.05 mm
*F*(000) = 2592	

### Data collection

**Table:**

Bruker SMART APEX CCD diffractometer	7415 independent reflections
Radiation source: fine-focus sealed tube	5577 reflections with *I* > 2σ(*I*)
Graphite monochromator	*R*_int_ = 0.086
Detector resolution: 8.3660 pixels mm^-1^	θ_max_ = 28.8°, θ_min_ = 2.0°
φ and ω scans	*h* = −22→22
Absorption correction: multi-scan (*SADABS*; Bruker, 2013)	*k* = −10→10
*T*_min_ = 0.80, *T*_max_ = 0.98	*l* = −55→56
99305 measured reflections	

### Refinement

**Table:**

Refinement on *F*^2^	Primary atom site location: structure-invariant direct methods
Least-squares matrix: full	Secondary atom site location: difference Fourier map
*R*[*F*^2^ > 2σ(*F*^2^)] = 0.044	Hydrogen site location: inferred from neighbouring sites
*wR*(*F*^2^) = 0.109	H-atom parameters constrained
*S* = 1.04	*w* = 1/[σ^2^(*F*_o_^2^) + (0.0371*P*)^2^ + 4.8434*P*] where *P* = (*F*_o_^2^ + 2*F*_c_^2^)/3
7415 reflections	(Δ/σ)_max_ = 0.001
379 parameters	Δρ_max_ = 0.42 e Å^−^^3^
0 restraints	Δρ_min_ = −0.33 e Å^−^^3^

### Special details

**Table:**

Experimental. The diffraction data were obtained from 3 sets of 400 frames, each of width 0.5° in ω, collected at φ = 0.00, 90.00 and 180.00° and 2 sets of 800 frames, each of width 0.45° in φ, collected at ω = -30.00 and 210.00°. The scan time was 15 sec/frame.
Geometry. All e.s.d.'s (except the e.s.d. in the dihedral angle between two l.s. planes) are estimated using the full covariance matrix. The cell e.s.d.'s are taken into account individually in the estimation of e.s.d.'s in distances, angles and torsion angles; correlations between e.s.d.'s in cell parameters are only used when they are defined by crystal symmetry. An approximate (isotropic) treatment of cell e.s.d.'s is used for estimating e.s.d.'s involving l.s. planes.
Refinement. Refinement of *F*^2^ against ALL reflections. The weighted *R*-factor *wR* and goodness of fit *S* are based on *F*^2^, conventional *R*-factors *R* are based on *F*, with *F* set to zero for negative *F*^2^. The threshold expression of *F*^2^ > σ(*F*^2^) is used only for calculating *R*-factors(gt) *etc*. and is not relevant to the choice of reflections for refinement. *R*-factors based on *F*^2^ are statistically about twice as large as those based on *F*, and *R*- factors based on ALL data will be even larger. H-atoms attached to carbon were placed in calculated positions (C—H = 0.95 Å) while those attached to nitrogen were placed in locations derived from a difference map and, following initial independent refinement to verify their presence, their coordinates were adjusted to give N—H = 0.91 Å. All were included as riding contributions with isotropic displacement parameters 1.2 - 1.5 times those of the attached atoms.

### Fractional atomic coordinates and isotropic or equivalent isotropic displacement parameters (Å^2^)

**Table:**

	*x*	*y*	*z*	*U*_iso_*/*U*_eq_	
Cl1	0.42874 (3)	0.85456 (8)	0.45306 (2)	0.04262 (15)	
S1	0.67429 (3)	0.24168 (6)	0.37657 (2)	0.02704 (12)	
N1	0.57267 (10)	0.4806 (2)	0.40738 (4)	0.0248 (3)	
N2	0.53242 (10)	0.3466 (2)	0.39636 (4)	0.0266 (4)	
H2A	0.4789	0.3475	0.3972	0.032*	
N3	0.54083 (9)	0.0943 (2)	0.36976 (4)	0.0235 (3)	
C1	0.52954 (12)	0.8719 (3)	0.44674 (5)	0.0266 (4)	
C2	0.56720 (14)	1.0112 (3)	0.45834 (5)	0.0325 (5)	
H2	0.5382	1.0943	0.4692	0.039*	
C3	0.64723 (14)	1.0279 (3)	0.45392 (5)	0.0354 (5)	
H3	0.6737	1.1225	0.4620	0.042*	
C4	0.68913 (13)	0.9078 (3)	0.43785 (5)	0.0326 (5)	
H4	0.7442	0.9205	0.4346	0.039*	
C5	0.65150 (12)	0.7700 (3)	0.42652 (5)	0.0268 (4)	
H5	0.6811	0.6881	0.4156	0.032*	
C6	0.57031 (11)	0.7473 (2)	0.43076 (4)	0.0229 (4)	
C7	0.53147 (11)	0.5992 (2)	0.41884 (5)	0.0244 (4)	
H7	0.4757	0.5905	0.4196	0.029*	
C8	0.57334 (11)	0.2272 (2)	0.38144 (4)	0.0221 (4)	
C9	0.67232 (11)	0.0512 (2)	0.35830 (5)	0.0263 (4)	
H9	0.7175	−0.0043	0.3503	0.032*	
C10	0.59793 (11)	−0.0091 (2)	0.35689 (4)	0.0226 (4)	
C11	0.57380 (11)	−0.1716 (2)	0.34490 (5)	0.0239 (4)	
C12	0.62690 (12)	−0.2731 (3)	0.32880 (5)	0.0295 (4)	
H12	0.6792	−0.2358	0.3252	0.035*	
C13	0.60464 (13)	−0.4266 (3)	0.31801 (5)	0.0339 (5)	
H13	0.6417	−0.4947	0.3073	0.041*	
C14	0.52845 (14)	−0.4821 (3)	0.32276 (5)	0.0336 (5)	
H14	0.5131	−0.5877	0.3151	0.040*	
C15	0.47497 (13)	−0.3835 (3)	0.33864 (5)	0.0321 (5)	
H15	0.4225	−0.4210	0.3418	0.039*	
C16	0.49763 (12)	−0.2292 (3)	0.34997 (5)	0.0276 (4)	
H16	0.4608	−0.1627	0.3613	0.033*	
Cl2	0.51373 (3)	−0.18369 (8)	0.25484 (2)	0.03684 (14)	
S2	0.23921 (3)	0.26852 (7)	0.35529 (2)	0.03059 (13)	
N4	0.35122 (10)	0.0848 (2)	0.31580 (4)	0.0254 (4)	
N5	0.38660 (10)	0.1670 (2)	0.34063 (4)	0.0279 (4)	
H5A	0.4365	0.1386	0.3464	0.033*	
N6	0.36410 (9)	0.2951 (2)	0.39001 (4)	0.0242 (3)	
C17	0.41198 (12)	−0.1953 (3)	0.25184 (5)	0.0255 (4)	
C18	0.38063 (13)	−0.2967 (3)	0.22837 (5)	0.0301 (4)	
H18	0.4145	−0.3575	0.2146	0.036*	
C19	0.30033 (13)	−0.3091 (3)	0.22512 (5)	0.0317 (5)	
H19	0.2786	−0.3785	0.2090	0.038*	
C20	0.25097 (13)	−0.2208 (3)	0.24522 (5)	0.0327 (5)	
H20	0.1955	−0.2294	0.2429	0.039*	
C21	0.28249 (12)	−0.1202 (3)	0.26869 (5)	0.0287 (4)	
H21	0.2482	−0.0608	0.2825	0.034*	
C22	0.36410 (12)	−0.1042 (2)	0.27246 (5)	0.0245 (4)	
C23	0.39660 (12)	−0.0014 (3)	0.29797 (5)	0.0258 (4)	
H23	0.4519	0.0014	0.3014	0.031*	
C24	0.33820 (11)	0.2402 (2)	0.36249 (5)	0.0232 (4)	
C25	0.23213 (12)	0.3603 (3)	0.39246 (5)	0.0299 (5)	
H25	0.1847	0.4030	0.4013	0.036*	
C26	0.30284 (11)	0.3639 (2)	0.40748 (5)	0.0236 (4)	
C27	0.31902 (11)	0.4215 (2)	0.44032 (5)	0.0233 (4)	
C28	0.26964 (12)	0.5346 (3)	0.45548 (5)	0.0281 (4)	
H28	0.2254	0.5774	0.4443	0.034*	
C29	0.28430 (13)	0.5849 (3)	0.48644 (5)	0.0325 (5)	
H29	0.2504	0.6627	0.4964	0.039*	
C30	0.34853 (14)	0.5223 (3)	0.50318 (5)	0.0353 (5)	
H30	0.3586	0.5568	0.5246	0.042*	
C31	0.39759 (13)	0.4097 (3)	0.48843 (5)	0.0334 (5)	
H31	0.4415	0.3667	0.4998	0.040*	
C32	0.38344 (12)	0.3590 (3)	0.45732 (5)	0.0269 (4)	
H32	0.4176	0.2813	0.4474	0.032*	

### Atomic displacement parameters (Å^2^)

**Table:**

	*U*^11^	*U*^22^	*U*^33^	*U*^12^	*U*^13^	*U*^23^
Cl1	0.0279 (3)	0.0448 (3)	0.0552 (4)	0.0053 (2)	0.0023 (2)	−0.0128 (3)
S1	0.0191 (2)	0.0260 (2)	0.0360 (3)	−0.00266 (19)	−0.00276 (19)	0.0003 (2)
N1	0.0269 (8)	0.0228 (8)	0.0246 (8)	−0.0049 (7)	−0.0031 (7)	−0.0022 (7)
N2	0.0205 (8)	0.0251 (9)	0.0341 (9)	−0.0045 (7)	0.0005 (7)	−0.0074 (7)
N3	0.0202 (7)	0.0236 (8)	0.0266 (8)	−0.0010 (6)	0.0007 (6)	−0.0024 (7)
C1	0.0280 (10)	0.0266 (10)	0.0253 (10)	0.0033 (8)	−0.0039 (8)	−0.0001 (8)
C2	0.0422 (12)	0.0242 (10)	0.0311 (11)	0.0037 (9)	−0.0050 (9)	−0.0056 (9)
C3	0.0445 (13)	0.0260 (11)	0.0356 (12)	−0.0073 (10)	−0.0116 (10)	−0.0035 (9)
C4	0.0326 (11)	0.0320 (11)	0.0333 (11)	−0.0066 (9)	−0.0033 (9)	0.0002 (9)
C5	0.0284 (10)	0.0275 (10)	0.0246 (10)	−0.0028 (8)	0.0002 (8)	−0.0012 (8)
C6	0.0272 (9)	0.0230 (9)	0.0184 (9)	−0.0020 (8)	−0.0031 (7)	0.0003 (7)
C7	0.0237 (9)	0.0258 (10)	0.0236 (9)	−0.0026 (8)	−0.0019 (7)	−0.0011 (8)
C8	0.0195 (8)	0.0245 (9)	0.0224 (9)	−0.0010 (7)	−0.0012 (7)	0.0006 (7)
C9	0.0227 (9)	0.0267 (10)	0.0295 (10)	0.0019 (8)	0.0008 (8)	0.0025 (8)
C10	0.0232 (9)	0.0243 (10)	0.0202 (9)	0.0030 (7)	−0.0015 (7)	0.0023 (7)
C11	0.0268 (9)	0.0233 (10)	0.0214 (9)	0.0014 (8)	−0.0041 (7)	0.0015 (7)
C12	0.0293 (10)	0.0300 (11)	0.0292 (10)	0.0032 (9)	−0.0006 (8)	−0.0032 (9)
C13	0.0391 (12)	0.0302 (11)	0.0325 (12)	0.0065 (9)	0.0008 (9)	−0.0042 (9)
C14	0.0454 (13)	0.0241 (10)	0.0313 (11)	−0.0014 (9)	−0.0036 (10)	−0.0025 (9)
C15	0.0342 (11)	0.0287 (11)	0.0334 (11)	−0.0042 (9)	−0.0025 (9)	0.0030 (9)
C16	0.0300 (10)	0.0249 (10)	0.0278 (10)	0.0002 (8)	−0.0001 (8)	−0.0002 (8)
Cl2	0.0278 (3)	0.0471 (3)	0.0356 (3)	0.0053 (2)	−0.0013 (2)	−0.0076 (2)
S2	0.0236 (2)	0.0383 (3)	0.0298 (3)	0.0043 (2)	−0.00573 (19)	−0.0034 (2)
N4	0.0273 (8)	0.0264 (9)	0.0226 (8)	−0.0035 (7)	−0.0023 (6)	−0.0020 (7)
N5	0.0219 (8)	0.0343 (10)	0.0274 (9)	0.0002 (7)	−0.0024 (7)	−0.0091 (7)
N6	0.0212 (8)	0.0261 (9)	0.0254 (8)	−0.0010 (7)	0.0002 (6)	−0.0030 (7)
C17	0.0278 (10)	0.0262 (10)	0.0227 (9)	0.0019 (8)	−0.0022 (7)	0.0030 (8)
C18	0.0400 (12)	0.0246 (10)	0.0257 (10)	0.0031 (9)	−0.0001 (9)	−0.0003 (8)
C19	0.0420 (12)	0.0261 (10)	0.0270 (11)	−0.0054 (9)	−0.0074 (9)	−0.0020 (9)
C20	0.0304 (10)	0.0339 (11)	0.0337 (11)	−0.0046 (9)	−0.0068 (9)	0.0008 (9)
C21	0.0297 (10)	0.0290 (11)	0.0275 (11)	0.0003 (9)	−0.0007 (8)	0.0000 (9)
C22	0.0295 (10)	0.0228 (10)	0.0211 (9)	−0.0003 (8)	−0.0027 (8)	0.0024 (8)
C23	0.0253 (9)	0.0281 (10)	0.0238 (10)	−0.0016 (8)	−0.0012 (7)	0.0007 (8)
C24	0.0204 (9)	0.0232 (9)	0.0259 (9)	−0.0020 (7)	−0.0010 (7)	−0.0002 (8)
C25	0.0241 (10)	0.0354 (12)	0.0302 (11)	0.0075 (9)	−0.0012 (8)	−0.0008 (9)
C26	0.0233 (9)	0.0203 (9)	0.0272 (10)	0.0006 (7)	0.0012 (7)	0.0013 (8)
C27	0.0241 (9)	0.0224 (9)	0.0234 (9)	−0.0015 (8)	0.0024 (7)	0.0014 (8)
C28	0.0284 (10)	0.0263 (10)	0.0296 (11)	0.0010 (8)	0.0011 (8)	0.0013 (9)
C29	0.0386 (12)	0.0287 (11)	0.0304 (11)	0.0028 (9)	0.0056 (9)	−0.0021 (9)
C30	0.0463 (13)	0.0361 (12)	0.0235 (10)	−0.0012 (10)	0.0006 (9)	−0.0017 (9)
C31	0.0348 (11)	0.0369 (12)	0.0287 (11)	0.0022 (9)	−0.0025 (9)	0.0020 (9)
C32	0.0256 (9)	0.0283 (10)	0.0269 (10)	0.0029 (8)	0.0014 (8)	−0.0002 (8)

### Geometric parameters (Å, º)

**Table:**

Cl1—C1	1.737 (2)	Cl2—C17	1.735 (2)
S1—C9	1.722 (2)	S2—C25	1.722 (2)
S1—C8	1.7301 (19)	S2—C24	1.723 (2)
N1—C7	1.282 (3)	N4—C23	1.278 (3)
N1—N2	1.363 (2)	N4—N5	1.370 (2)
N2—C8	1.344 (2)	N5—C24	1.363 (2)
N2—H2A	0.9098	N5—H5A	0.9100
N3—C8	1.305 (2)	N6—C24	1.306 (2)
N3—C10	1.389 (2)	N6—C26	1.387 (2)
C1—C2	1.385 (3)	C17—C18	1.384 (3)
C1—C6	1.394 (3)	C17—C22	1.395 (3)
C2—C3	1.378 (3)	C18—C19	1.374 (3)
C2—H2	0.9500	C18—H18	0.9500
C3—C4	1.380 (3)	C19—C20	1.384 (3)
C3—H3	0.9500	C19—H19	0.9500
C4—C5	1.371 (3)	C20—C21	1.382 (3)
C4—H4	0.9500	C20—H20	0.9500
C5—C6	1.402 (3)	C21—C22	1.401 (3)
C5—H5	0.9500	C21—H21	0.9500
C6—C7	1.458 (3)	C22—C23	1.459 (3)
C7—H7	0.9500	C23—H23	0.9500
C9—C10	1.356 (3)	C25—C26	1.354 (3)
C9—H9	0.9500	C25—H25	0.9500
C10—C11	1.468 (3)	C26—C27	1.472 (3)
C11—C16	1.391 (3)	C27—C28	1.394 (3)
C11—C12	1.393 (3)	C27—C32	1.398 (3)
C12—C13	1.376 (3)	C28—C29	1.375 (3)
C12—H12	0.9500	C28—H28	0.9500
C13—C14	1.384 (3)	C29—C30	1.390 (3)
C13—H13	0.9500	C29—H29	0.9500
C14—C15	1.379 (3)	C30—C31	1.380 (3)
C14—H14	0.9500	C30—H30	0.9500
C15—C16	1.391 (3)	C31—C32	1.381 (3)
C15—H15	0.9500	C31—H31	0.9500
C16—H16	0.9500	C32—H32	0.9500
			
C9—S1—C8	88.38 (9)	C25—S2—C24	88.24 (9)
C7—N1—N2	116.74 (16)	C23—N4—N5	116.15 (17)
C8—N2—N1	118.06 (16)	C24—N5—N4	116.89 (16)
C8—N2—H2A	122.8	C24—N5—H5A	119.7
N1—N2—H2A	118.7	N4—N5—H5A	118.9
C8—N3—C10	110.30 (16)	C24—N6—C26	110.19 (16)
C2—C1—C6	121.89 (19)	C18—C17—C22	121.71 (19)
C2—C1—Cl1	117.90 (16)	C18—C17—Cl2	117.79 (16)
C6—C1—Cl1	120.21 (16)	C22—C17—Cl2	120.50 (15)
C3—C2—C1	119.3 (2)	C19—C18—C17	119.6 (2)
C3—C2—H2	120.4	C19—C18—H18	120.2
C1—C2—H2	120.4	C17—C18—H18	120.2
C2—C3—C4	120.3 (2)	C18—C19—C20	120.2 (2)
C2—C3—H3	119.9	C18—C19—H19	119.9
C4—C3—H3	119.9	C20—C19—H19	119.9
C5—C4—C3	120.1 (2)	C21—C20—C19	119.9 (2)
C5—C4—H4	119.9	C21—C20—H20	120.0
C3—C4—H4	119.9	C19—C20—H20	120.0
C4—C5—C6	121.5 (2)	C20—C21—C22	121.2 (2)
C4—C5—H5	119.3	C20—C21—H21	119.4
C6—C5—H5	119.3	C22—C21—H21	119.4
C1—C6—C5	116.98 (18)	C17—C22—C21	117.30 (18)
C1—C6—C7	122.36 (18)	C17—C22—C23	122.05 (18)
C5—C6—C7	120.66 (18)	C21—C22—C23	120.59 (18)
N1—C7—C6	119.91 (18)	N4—C23—C22	120.50 (18)
N1—C7—H7	120.0	N4—C23—H23	119.8
C6—C7—H7	120.0	C22—C23—H23	119.8
N3—C8—N2	123.26 (17)	N6—C24—N5	122.14 (17)
N3—C8—S1	115.56 (15)	N6—C24—S2	115.85 (15)
N2—C8—S1	121.18 (14)	N5—C24—S2	122.01 (14)
C10—C9—S1	111.14 (15)	C26—C25—S2	111.26 (15)
C10—C9—H9	124.4	C26—C25—H25	124.4
S1—C9—H9	124.4	S2—C25—H25	124.4
C9—C10—N3	114.57 (18)	C25—C26—N6	114.46 (18)
C9—C10—C11	126.78 (18)	C25—C26—C27	126.99 (18)
N3—C10—C11	118.58 (17)	N6—C26—C27	118.41 (17)
C16—C11—C12	118.49 (19)	C28—C27—C32	118.67 (19)
C16—C11—C10	120.61 (18)	C28—C27—C26	121.19 (18)
C12—C11—C10	120.88 (18)	C32—C27—C26	120.11 (18)
C13—C12—C11	120.9 (2)	C29—C28—C27	120.7 (2)
C13—C12—H12	119.6	C29—C28—H28	119.6
C11—C12—H12	119.6	C27—C28—H28	119.6
C12—C13—C14	120.3 (2)	C28—C29—C30	120.3 (2)
C12—C13—H13	119.9	C28—C29—H29	119.9
C14—C13—H13	119.9	C30—C29—H29	119.9
C15—C14—C13	119.7 (2)	C31—C30—C29	119.4 (2)
C15—C14—H14	120.1	C31—C30—H30	120.3
C13—C14—H14	120.1	C29—C30—H30	120.3
C14—C15—C16	120.1 (2)	C30—C31—C32	120.7 (2)
C14—C15—H15	119.9	C30—C31—H31	119.7
C16—C15—H15	119.9	C32—C31—H31	119.7
C11—C16—C15	120.5 (2)	C31—C32—C27	120.23 (19)
C11—C16—H16	119.8	C31—C32—H32	119.9
C15—C16—H16	119.8	C27—C32—H32	119.9
			
C7—N1—N2—C8	173.77 (18)	C23—N4—N5—C24	−170.95 (18)
C6—C1—C2—C3	0.2 (3)	C22—C17—C18—C19	−0.1 (3)
Cl1—C1—C2—C3	179.50 (17)	Cl2—C17—C18—C19	−179.82 (17)
C1—C2—C3—C4	0.6 (3)	C17—C18—C19—C20	−0.1 (3)
C2—C3—C4—C5	−0.8 (3)	C18—C19—C20—C21	−0.2 (3)
C3—C4—C5—C6	0.2 (3)	C19—C20—C21—C22	0.6 (3)
C2—C1—C6—C5	−0.8 (3)	C18—C17—C22—C21	0.5 (3)
Cl1—C1—C6—C5	179.94 (15)	Cl2—C17—C22—C21	−179.80 (15)
C2—C1—C6—C7	179.12 (19)	C18—C17—C22—C23	177.84 (19)
Cl1—C1—C6—C7	−0.1 (3)	Cl2—C17—C22—C23	−2.4 (3)
C4—C5—C6—C1	0.6 (3)	C20—C21—C22—C17	−0.7 (3)
C4—C5—C6—C7	−179.34 (19)	C20—C21—C22—C23	−178.12 (19)
N2—N1—C7—C6	−179.33 (17)	N5—N4—C23—C22	175.08 (17)
C1—C6—C7—N1	−172.59 (19)	C17—C22—C23—N4	177.70 (19)
C5—C6—C7—N1	7.3 (3)	C21—C22—C23—N4	−5.0 (3)
C10—N3—C8—N2	−177.70 (18)	C26—N6—C24—N5	179.78 (18)
C10—N3—C8—S1	2.3 (2)	C26—N6—C24—S2	0.6 (2)
N1—N2—C8—N3	−179.50 (18)	N4—N5—C24—N6	167.05 (18)
N1—N2—C8—S1	0.5 (3)	N4—N5—C24—S2	−13.8 (3)
C9—S1—C8—N3	−1.55 (16)	C25—S2—C24—N6	−0.41 (17)
C9—S1—C8—N2	178.42 (17)	C25—S2—C24—N5	−179.58 (18)
C8—S1—C9—C10	0.33 (16)	C24—S2—C25—C26	0.07 (17)
S1—C9—C10—N3	0.9 (2)	S2—C25—C26—N6	0.3 (2)
S1—C9—C10—C11	−175.89 (16)	S2—C25—C26—C27	−175.32 (16)
C8—N3—C10—C9	−2.0 (2)	C24—N6—C26—C25	−0.6 (3)
C8—N3—C10—C11	175.04 (17)	C24—N6—C26—C27	175.43 (17)
C9—C10—C11—C16	168.6 (2)	C25—C26—C27—C28	−24.5 (3)
N3—C10—C11—C16	−8.0 (3)	N6—C26—C27—C28	160.09 (18)
C9—C10—C11—C12	−10.1 (3)	C25—C26—C27—C32	153.4 (2)
N3—C10—C11—C12	173.30 (18)	N6—C26—C27—C32	−22.0 (3)
C16—C11—C12—C13	0.2 (3)	C32—C27—C28—C29	0.6 (3)
C10—C11—C12—C13	178.94 (19)	C26—C27—C28—C29	178.54 (19)
C11—C12—C13—C14	0.6 (3)	C27—C28—C29—C30	−0.5 (3)
C12—C13—C14—C15	−0.6 (3)	C28—C29—C30—C31	0.2 (3)
C13—C14—C15—C16	−0.3 (3)	C29—C30—C31—C32	0.0 (3)
C12—C11—C16—C15	−1.2 (3)	C30—C31—C32—C27	0.1 (3)
C10—C11—C16—C15	−179.87 (18)	C28—C27—C32—C31	−0.4 (3)
C14—C15—C16—C11	1.2 (3)	C26—C27—C32—C31	−178.36 (19)

### Hydrogen-bond geometry (Å, º)

**Table:**

*D*—H···*A*	*D*—H	H···*A*	*D*···*A*	*D*—H···*A*
N2—H2*A*···N6	0.91	2.02	2.901 (2)	163
N5—H5*A*···N3	0.91	2.05	2.946 (2)	166
C9—H9···S1^i^	0.95	2.97	3.696 (2)	134
C25—H25···Cl1^ii^	0.95	2.92	3.720 (2)	143

Symmetry codes: (i) −*x*+3/2, *y*−1/2, *z*; (ii) −*x*+1/2, *y*−1/2, *z*.

## References

[bb1] Bakris, G. L., Bank, A. J., Kass, D. A., Neutel, J. M., Preston, R. A. & Oparil, S. (2004). *Am. J. Hypertens.* **17**, 23S–30S. 10.1016/j.amjhyper.2004.08.022 15607432

[bb2] Brandenburg, K. & Putz, H. (2012). *DIAMOND* Crystal Impact GbR, Bonn, Germany.

[bb3] Bruker (2013). *APEX2*, *SHELXTL*, *SADABS* and *SAINT* Bruker AXS Inc., Madison, Wisconsin, USA.

[bb4] Little, W. C., Zile, M. R., Kitzman, D. W., Hundley, W. G., O’Brien, T. X. & Degroof, R. C. (2005). *J. Card. Fail.* **11**, 191–195. 10.1016/j.cardfail.2004.09.010 15812746

[bb5] Mohamed, S. K., Mague, J. T., Akkurt, M., Hassan, A. A. & Albayati, M. R. (2013). *Acta Cryst.* E**69**, o1324. 10.1107/S1600536813020254 PMC3793809 24109396

[bb6] Sheldrick, G. M. (2008). *Acta Cryst.* A**64**, 112–122. 10.1107/S0108767307043930 18156677

[bb7] Siddiqui, N., Arshad, M. F., Ahsan, W. & Alam, M. S. (2009). *IJPSDR*, **1**, 136–143.

[bb8] Siddiqui, N., Arya, S. K., Ahsan, W. & Azad, B. (2011). *Int. J. Drug Dev. Res.*, **3**, 55–67.

