# Supplementary figures and images for: Crystal structure of 2-[(E)-2-(2-chloro­benzyl­idene)hydrazin-1-yl]-4-phenyl-1,3-thia­zole
Source: Acta Crystallogr Sect E Struct Rep Online. 2014 Aug 1;70(Pt 9):o907–8. doi: 10.1107/S1600536814016298 (PMC4186150; doi:10.1107/S1600536814016298)

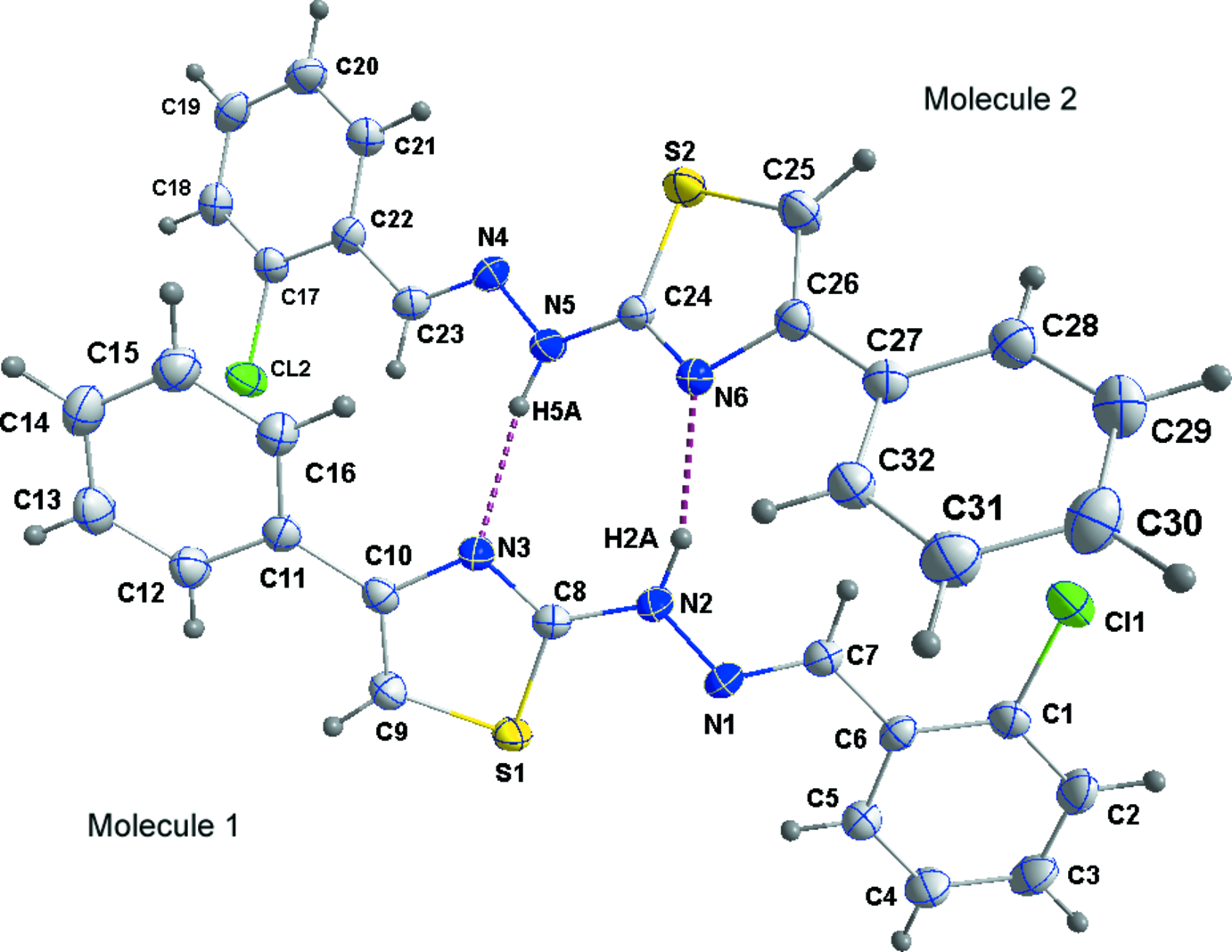

Supplement: Supplementary file 4 [file e-70-0o907-fig1.tif]
